# Orbital Signaling in Graves’ Orbitopathy

**DOI:** 10.3389/fendo.2021.739994

**Published:** 2021-11-09

**Authors:** Mohd Shazli Draman, Lei Zhang, Colin Dayan, Marian Ludgate

**Affiliations:** ^1^ Thyroid Research Group, Cardiff University School of Medicine, Cardiff, United Kingdom; ^2^ KPJ Healthcare University College, Nilai, Malaysia

**Keywords:** thyroid eye disease, adipogenesis, hyaluronan, TSAB, TSHR, IGF1R

## Abstract

Graves’ orbitopathy (GO) is a complex and poorly understood disease in which extensive remodeling of orbital tissue is dominated by adipogenesis and hyaluronan production. The resulting proptosis is disfiguring and underpins the majority of GO signs and symptoms. While there is strong evidence for the thyrotropin receptor (TSHR) being a thyroid/orbit shared autoantigen, the insulin-like growth factor 1 receptor (IGF1R) is also likely to play a key role in the disease. The pathogenesis of GO has been investigated extensively in the last decade with further understanding of some aspects of the disease. This is mainly derived by using *in vitro* and *ex vivo* analysis of the orbital tissues. Here, we have summarized the features of GO pathogenesis involving target autoantigens and their signaling pathways.

## Introduction

Graves’ orbitopathy (GO) or thyroid eye disease is the most common overt thyroidal manifestation of Graves’ disease (GD) with substantial morbidity and socioeconomic impact (1–4). Extensive orbital tissue remodelling in GO is mainly shown as adipose tissue expansion and tissue edema *via* increased adipogenesis and hyaluronan production, respectively. These pathogenetic processes produce disfiguring proptosis and underpin all GO signs and symptoms. There is a close clinical and temporal association between GD and GO suggesting an autoimmune response to common antigen/s in the orbit and thyroid gland. The thyrotropin receptor (TSHR) is expressed in orbital adipose tissue (OAT) (5–8) and virtually all patients with hyperthyroid GO have thyroid stimulating antibodies (TSAB). Therefore, the TSHR is the most logical candidate (9), which is further supported by the existence of TSHR-induced GO in an animal model (10). The incidence of GO is estimated to be 16/100,000 in females and 2.9/100,000 in males (11). On the other hand, the prevalence estimate is about 10/10,000 (12). A recent meta-analysis reported that current GD patients have a milder phenotype than in the past; as a consequence, a smaller proportion display GO symptoms (13). As with other autoimmune conditions there is female preponderance towards the condition with 6:1 female to male ratio, although in GO the ratio is less skewed than in GD. In addition, most patients with GO have reduced quality of life (QOL) (14) and suffer long-term psychological distress due to the disfiguring appearance of the proptosis, also known as exophthalmos (15). Available treatments for GO are unsatisfactory and more research is needed to address the pathophysiology of the disease which may lead to early pre-clinical diagnosis promoting preventative/early interventions. This in turn will improve long-term morbidity and socioeconomic impact.

## Adipogenesis

Adipogenesis is a process in which preadipocytes differentiate into mature adipocytes to form adipose tissues. Our current understanding of adipogenesis has been largely derived by using the murine 3T3L1 cell line. This cell line can spontaneously differentiate into adipocytes when maintained in a high concentration of fetal calf serum for several weeks but the process can be accelerated by employing adipogenic cocktails including insulin, steroid and 3- isobutyl-1-methylxanthine (IBMX) (16). Further components of the differentiation cocktails may also include proliferation-activated receptor gamma (PPARγ) agonists such as pioglitazone and indomethacin (17). Insulin, in common with insulin-like growth factor-1 (IGF-1) activates PI3 kinase (18) and MAP (mitogen-activated protein kinase) (19) pathways. Phosphorylation of protein kinase B (PKB/Akt) in turn phosphorylates forkhead box protein O1 (FOXO1) causing it to exit from the nucleus leading to increased transcription of adipogenic genes (20). Steroids induce the expression of the early adipogenic gene, CCAAT enhancer binding protein delta (C/EBP-δ). This transcription factor contributes to an increase in PPAR-γ expression and production of prostacyclin leading to elevated intracellular cAMP. IBMX is a nonselective phosphodiesterase inhibitor whose presence further elevates levels of intracellular cAMP and protein kinase A (PKA). IBMX is thus required for transcriptional activation of the master regulator of adipogenesis, PPARγ.

Adipogenesis contributes to OAT expansion because a fibroblast has an approximate diameter of 30 microns, whereas the diameter of a mature adipocyte is approximately 150 microns, i.e. 5 times larger. The increased adipogenesis has been demonstrated by using *in vitro* cultures of human fibroblasts and analysis of *ex vivo* samples from patients with GO (21). By using both *in vitro* lineage specific differentiation protocols and flow cytometry, studies have indicated that orbital fibroblasts (OF) possess mesenchymal stem cell (MSC) properties including positivity for Thy-1 (CD90) which is a marker of MSC (22–25). In the orbit, Thy-1 negative OF can be induced to differentiate when cultured in appropriate adipogenic medium whereas Thy-1 positive cells are more likely to undergo differentiation to myofibroblasts and cause fibrosis (23, 24). The orbital fibroblast is also able to undergo neurogenesis, myogenesis, osteogenesis and chondrogenesis *in vitro*, indicating their pluripotency (22, 25).

## Extra-Cellular Matrix

Several extracellular matrix (ECM) components are overproduced in GO including collagens and glycosaminoglycans (GAGs). The excess ECM accumulation in OAT and extraocular muscle (EOM) lead to oedema with consequent proptosis and diplopia respectively (26). The main GAG produced in GO is hyaluronic acid, which is generated by three synthase enzymes (HAS1, HAS2 and HAS3) and broken down by hyaluronidases. Activation of cAMP-protein kinase A signaling *via* the TSHR, increases cAMP response element binding protein (CREB) at CREB binding sites in the promoters of HAS1 and HAS2 genes, thereby enhancing hyaluronan production (27).

## TSHR Intracellular Pathways

Several studies, including from our group, have shown that activation of the TSHR in OF leads to an increase in hyaluronan production and adipogenesis (20, 28). TSHR expression has been shown to increase during adipogenesis (5). We demonstrated that ‘neutral’ TSHR antibodies were capable of binding but had no effect on traditional TSHR signaling pathways (described below) (29). Indeed, TSHR signaling may be far more complex than initially thought (30). Little is known about the effects of TSHR activation at various stages during differentiation. The downstream cascade triggered by TSHR will depend on the types and abundance of guanine-nucleotide binding proteins (G proteins) available in the cell (31). G protein coupled receptors (GPCR) can exist as monomers or oligomers. Oligomerization is the term used to describe dimeric, tetrameric, or higher-order complexes between GPCR monomers. The activation of different GPCR complexes will have major influence on subsequent G protein signaling pathways. The evidence that TSHR may exist in an oligomeric state was initially provided by studies using antibodies (32) and more recently by fluorescence resonance energy transfer (FRET) technology (33). Interestingly, the presence of dimerization influences TSHR behavior. Unstimulated TSHRs have been shown to form oligomers that return to the monomer state with TSH (34). TSHR autoantibodies with stimulating properties are (TSAB) proposed to favor formation of TSHR dimers, whilst TSHR blocking antibodies, are unable to bring about this conformational change. After TSH binding, a constitutively oligomeric TSHR dissociates into active monomers (or dimers when TSAB bind). Subsequently the monomers or dimers are recruited to the lipid rafts and interact with G proteins, thereby initiating the signaling cascade. In the case of TSH, the signal is rapid and brief because of faster movement of monomers into the lipid rafts, in contrast to the slow motion of the dimers. Multivalent blocking TSHR antibodies may cross-link the oligomers, thus preventing them from dissociating and impeding their entry into lipid rafts (35). In cells with low levels of TSHR expression, homo-heterodimer formation is less likely. This may change during adipogenesis, as TSHR expression increases, and may lead to activation of different signaling cascades from that predominating in orbital fibroblasts.

TSHR is known to activate mainly the guanine-nucleotide protein alpha stimulation (Gs)-cAMP pathway. In addition, TSHR may activate several other G protein subtypes, as detailed below (36, 37), non G protein pathways such as β-arrestin-1 (38) and other signalling pathways (39, 40). When TSH binds to its receptor, GTP replaces GDP in the heterotrimeric G protein, which dissociates into Gsα and Gβγ subunits with the former activating all isoforms of adenylate cyclase (41). This enzyme increases levels of cAMP in the cell and activates PKA, also known as cAMP-dependent protein kinase. The activated PKA phosphorylates multiple downstream target proteins one of which is cAMP responsive element binding protein (CREB). CREB then binds to its receptors on the promoter region of the DNA exerting various gene transcription processes including expression of thyroglobulin (TG), thyroid peroxidase (TPO), sodium iodide symporter (NIS), the thyroid transcription factors TTF1/NKx2.1, TTF2/FoxE1, and PAX (42, 43). Every intermediary in the pathway described above may additionally interact with different molecules belonging to other pathways.

In human thyrocytes and rat FRTL-5, guanidine binding protein alpha a/alpha 11 (Gαq/α11) coupling has been shown to stimulate Protein kinase C (PKC) pathways by generating phospholipase C (PLCβ). The PKC pathways has been associated with hyaluronan generation in GO (44). Activation of PKC pathways requires supraphysiological TSH concentrations although not all research agrees with this finding (45). PLC catalyses hydrolysis of phosphatidylinositol in cell membranes yielding di-acyl-glycerol (DAG) and inositol tri phosphate (IP3) as second messengers. DAG directly stimulates PKC. IP3 increases cytosolic Ca+2 levels which act through a number of effectors including PKC itself (46) and Nuclear Factor of Activated T-cells (NFAT) transcription factor protein. NFAT plays an important role in cytokine gene transcription regulation (47). Calcium *via* calmodulin –a calcium sensor protein - activates the serine/threonine phosphatase calcineurin (inhibited by cyclosporin and FK506). This in turn rapidly dephosphorylates NFAT proteins, resulting in a conformational change that exposes a nuclear localization signal leading to NFAT nuclear import (48). TSHR may also couple to guanine nucleotide binding protein alpha inhibition (Gαi1), which inhibits adenylyl cyclase and decreases cAMP levels. The accompanying Gβγ dimers may induce multitudes of other pathways, including adenylyl cyclase, PI3K/Akt (PKB)-FOXO and PLC cascades (49–51). Others have reported that TSHR activation of OF signals *via* p70s6 kinase (52). The finding may explain our lack of success when using gain-of-function mutants of the TSHR, which signal predominantly *via* Gsα, to stimulate adipogenesis (28) and concurs with the study from van Ziejl et al. who investigated TSH/TSAB induced hyaluronan production (53). It contrasts with the studies of Neumann and colleagues, who report increased M22-mediated cAMP, even at baseline. However, these authors maintain their OF in a semi-adipogenic medium which likely increases TSHR expression (54).

Our previous work has demonstrated that adipogenesis and HA production, are linked in the orbit. HA accumulation increases in the orbit during adipogenesis but not in other fat depots (55). In this study, adipogenesis in orbital preadipocytes was accompanied by HA accumulation and significantly increased *HAS2* transcripts (but not *HAS 1* and *3*). In contrast, adipogenic differentiation in subcutaneous preadipocyte-fibroblasts significantly decreased secreted HA and *HAS2* transcript levels. IGF-I alone did not increase *HAS2* levels, but inhibition of PKB/Akt increased orbital *HAS2* transcripts but not subcutaneous preadipocytes. Furthermore, our study suggested that mTORC1 negative feedback in IGF1–PI3K–Akt signalling is absent in OF but present in subcutaneous adipose tissue (55). The difference might be explained by the fact that human OF originate from neural crest, while subcutaneous adipose tissue is of mesodermal origin. In addition, our most recent studies demonstrated a depot specific fatty acid-uptake driven adipogenesis with unique gene signatures in OAT. These result in hyperplastic-type expansion of adipocytes in GO (56, 57). Taken together, these findings suggest a very distinctive mechanism underlying the orbital adipogenesis process.

## Insulin Like Growth Factor -1 Receptor Signalling

While there is strong evidence supporting the role of TSHR in GO, IGF1R is also likely to play a key role in the disease progress. The IGF1R was first proposed by Weightman and colleagues who demonstrated high affinity IGF1 binding sites in OF (58). More recently extensive work from Terry Smith and his colleagues has confirmed this finding and further showed that TSHR and IGF1R co-localize to orbital cell membranes (59). The same group has further reported a wide range of IGF1R mediated effects in OF including increases in proliferation, GAG production and cytokine production (60, 61). Our own study demonstrated that activation of TSHR and IGR1R has additive effect on HAS2 transcripts/HA production (62). Krieger et al. found that M22 stimulation of HA secretion by OF involves cross talk between IGF-1R and TSHR. The relationship relies on TSHR activation per se rather than direct activation of IGF-1R which leads to synergistic stimulation of HA secretion (63). TSH induced ERK phosphorylation can be blocked by an IGF-1R-blocking monoclonal antibody suggesting that IGF-1R might mediate some TSH-provoked signalling. Further studies have highlighted the importance of down stream factors of IGF1–PI3K signalling and revealed that FOXOs, may mediate both TSHR and IGF1R signalling pathways in GO (64). The notion is further supported by recent successful trial of teprotumumab- monoclonal antibody which blocks IGF1R - in reducing proptosis in patients with GO (65, 66). Whilst effective medical treatment for GO is welcome, some concerns have been raised about these trials including the lack of orbital imaging and the fact that despite QOL scores being improved in the teprotumumab group, all patients scores remained low (67). Furthermore, the activation of Fibroblast Growth factor (FGF) and its receptor has been shown to increase the expression of Insulin like growth factor-2 (IGF-2) in mesenchymal stem cells *via* IGF-2 and IGF1-R (68). The FGF signalling pathway has also been shown to play a role in OAT expansion in GO (69). Our most recent study used RNA-seq analysis to demonstrate that FGFs, FGFR2, IGF-2 and IGF1-R were highly expressed in OAT compared with white adipose tissue, supporting the aforementioned successful trial of IGF1R inhibition in GO (56).

## TSHR Variants

To add to the complexity of the molecular events associated with GO, several TSHR variants have been described which lack the transmembrane domain. If the variants are expressed as protein, they would yield soluble receptor products which could serve as TSH/TRAB binding proteins or even as autoantigens. Early northern blot analysis of thyroid tissue identified the expected full-length transcript plus 2 additional transcripts at 1.3 and 1.6 kb (70); the transcripts were also detected in OF (71). Of interest, the exon 1-8 variant is similar in structure to the TSHR A subunit which is generated following cleavage of the full-length receptor (72, 73). Furthermore, induced murine models of GD and GO are more effective when immunizing with the A subunit than with the complete TSHR (74, 75). We have reported that the 1.3 variant is expressed as a protein and can affect TSHR activation (76). Thus, these variants could have impact on the pathogenesis of GO by inducing further production of TSAB or protect against GO by ‘neutralizing’ TSAB, respectively.

## Discussion

**Figure 1:**
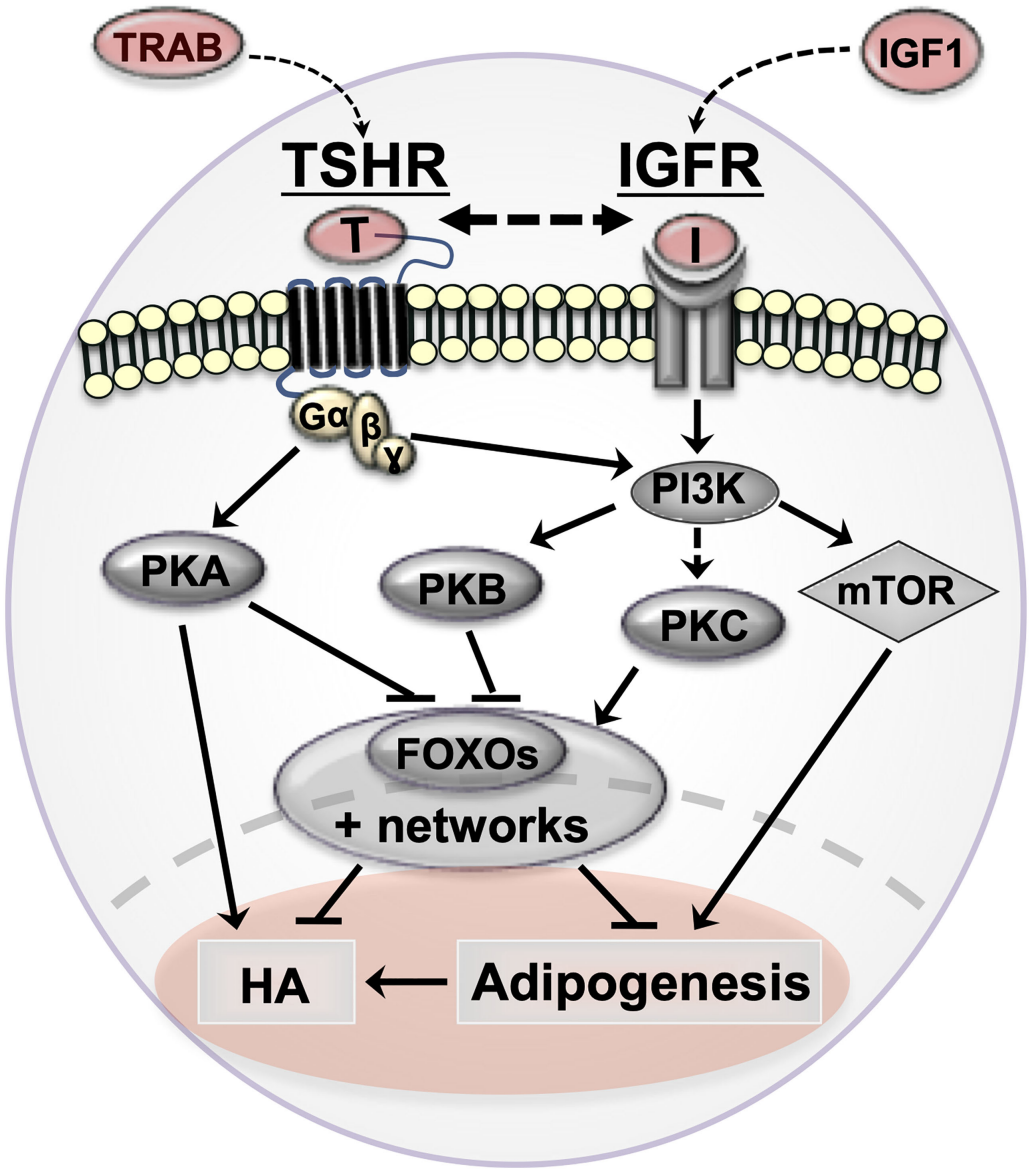
Cartoon summarizing orbital fibroblast signaling cascades in Graves’ orbitopathy (GO) and how they affect pathogenetic mechanisms (adipogenesis and hyaluronan production). TSHR/TRAB and IGFR/IGF are shown in red with arrows indicating the possible crosstalk between the pathways in GO. thyrotropin receptor (TSHR, serpentine structure); TSHR auto-antibodies (TRAB); Insulin-like growth factor 1 receptor (IGF-1R) and IGF1; protein kinase A (PKA); protein kinase B (PKB/Akt); protein kinase C (PKC); phosphoinositide 3-kinase (PI3K); forkhead box protein O (FOXO); hyaluronan production (HA).

TSHR and IGF1 signaling are important in orbital tissues (summarized in **Figure 1**) but more complex than generally thought. Although these signals are mainly activated through G protein signalling pathways, other cascades may also be involved. As our understanding expands, additional extracellular or intracellular factors, which regulate signaling, may be identified. The abundance of the receptors may also dictate which pathways are activated. The recent success of TSHR extracellular domain crystallization is likely to catapult these areas of research and may lead to further alternative treatment strategies for GO (77).

As discussed above, a human monoclonal anti-IGF-1R-blocking antibody, Teprotumumab has been approved by FDA for treatment of patients with GO specifically in reducing proptosis and has recently been reported to be highly effective in active GO (65). The potential for treatments based on TSHR antagonism, which have been demonstrated to be effective *in vitro*, is keenly anticipated either with blocking antibodies or small molecule antagonists which in theory could inhibit both TSHR and IGF-1R related and/or unrelated pathways (78). The beneficial effects on GD and GO following administration of a monoclonal TSHR blocking antibody (TBAB) in a patient with thyroid cancer has recently been described (79). Furthermore, manipulating the two pathways concomitantly may provide even more effective treatment for GO and merits investigation.

## Author Contributions

MD and LZ wrote the manuscript with input from ML. CD and ML reviewed the manuscript. All authors contributed to the article and approved the submitted version.

## Conflict of Interest

The authors declare that the research was conducted in the absence of any commercial or financial relationships that could be construed as a potential conflict of interest.

## Publisher’s Note

All claims expressed in this article are solely those of the authors and do not necessarily represent those of their affiliated organizations, or those of the publisher, the editors and the reviewers. Any product that may be evaluated in this article, or claim that may be made by its manufacturer, is not guaranteed or endorsed by the publisher.

## Abbreviations

GO, Graves’ orbitopathy; TSAB, thyroid stimulating antibodies; TSHR, thyrotropin receptor; IGF1, insulin like growth factor 1; IBMX, 3-isobutyl-1-methylxanthine; PPARγ, peroxisome proliferator-activated receptors gamma; PI3 kinase Phosphoinositide-3- kinase; MAP, mitogen-activated protein kinase; FOXO, Forkhead Box O1; C/EBP-δ, CCAAT-enhancer-binding proteins Delta; cAMP, Cyclic adenosine monophosphate; PKA, protein kinase A; GCRP, G protein coupled receptors; FRET, fluorescence resonance energy transfer; CREB, cAMP responsive element binding protein; TG, thyroglobulin; TPO, thyroid peroxidase; NIS, sodium iodide symporter (NIS), TTF, thyroid transcription factors; PKC, Protein kinase C; PLC phospholipase C; DAG, di-acyl-glycerol; NFAT, Nuclear factor of activated T-cells; HA, hyaluronic acid; GAG, glycosaminoglycan; SNP, single nucleotide polymorphism.
